# CaeNDR, the *Caenorhabditis* Natural Diversity Resource

**DOI:** 10.1093/nar/gkad887

**Published:** 2023-10-19

**Authors:** Timothy A Crombie, Ryan McKeown, Nicolas D Moya, Kathryn S Evans, Samuel J Widmayer, Vincent LaGrassa, Natalie Roman, Orzu Tursunova, Gaotian Zhang, Sophia B Gibson, Claire M Buchanan, Nicole M Roberto, Rodolfo Vieira, Robyn E Tanny, Erik C Andersen

**Affiliations:** Department of Biomedical and Chemical Engineering and Sciences, Florida Institute of Technology, Melbourne, FL, USA; Department of Molecular Biosciences, Northwestern University, Evanston, IL, USA; Department of Molecular Biosciences, Northwestern University, Evanston, IL, USA; Interdisciplinary Biological Sciences Program, Northwestern University, Evanston, IL, USA; Department of Biology, Johns Hopkins University, Baltimore, MD, USA; Cell, Molecular, Developmental biology, and Biophysics Graduate Program, ohns Hopkins University, Baltimore, MD, USA; Department of Molecular Biosciences, Northwestern University, Evanston, IL, USA; Department of Molecular Biosciences, Northwestern University, Evanston, IL, USA; Northwestern University Information Technology, Media and Technology Innovation, Northwestern University, Evanston, IL USA; Northwestern University Information Technology, Media and Technology Innovation, Northwestern University, Evanston, IL USA; Northwestern University Information Technology, Media and Technology Innovation, Northwestern University, Evanston, IL USA; Department of Molecular Biosciences, Northwestern University, Evanston, IL, USA; Department of Molecular Biosciences, Northwestern University, Evanston, IL, USA; Department of Molecular Biosciences, Northwestern University, Evanston, IL, USA; Department of Molecular Biosciences, Northwestern University, Evanston, IL, USA; Northwestern University Information Technology, Media and Technology Innovation, Northwestern University, Evanston, IL USA; Department of Biology, Johns Hopkins University, Baltimore, MD, USA; Department of Biology, Johns Hopkins University, Baltimore, MD, USA

## Abstract

Studies of model organisms have provided important insights into how natural genetic differences shape trait variation. These discoveries are driven by the growing availability of genomes and the expansive experimental toolkits afforded to researchers using these species. For example, *Caenorhabditis elegans* is increasingly being used to identify and measure the effects of natural genetic variants on traits using quantitative genetics. Since 2016, the *C. elegans* Natural Diversity Resource (CeNDR) has facilitated many of these studies by providing an archive of wild strains, genome-wide sequence and variant data for each strain, and a genome-wide association (GWA) mapping portal for the *C. elegans* community. Here, we present an updated platform, the *Caenorhabditis* Natural Diversity Resource (CaeNDR), that enables quantitative genetics and genomics studies across the three *Caenorhabditis* species: *C. elegans*, *C. briggsae* and *C. tropicalis*. The CaeNDR platform hosts several databases that are continually updated by the addition of new strains, whole-genome sequence data and annotated variants. Additionally, CaeNDR provides new interactive tools to explore natural variation and enable GWA mappings. All CaeNDR data and tools are accessible through a freely available web portal located at caendr.org.

## Graphical Abstract

**Graphical Abstract:**
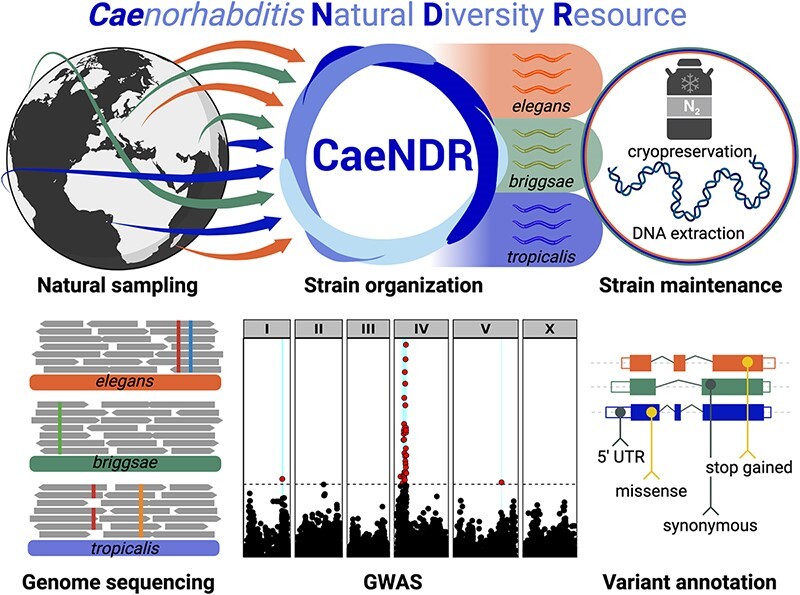

## Introduction

*Caenorhabditis elegans* is regarded as a powerful model because of its experimental tractability, which has facilitated major contributions to the fields of developmental, cellular and neurobiology. *C. elegans* is a small (∼1 mm), free-living nematode that has a near-global distribution and is most often found in association with decomposing plant material (1). Several attributes make *C. elegans* an exceptional animal model. First, the species has a short generation time of approximately four days, wherein an individual produces hundreds of offspring (2). Its genome is highly compact (∼100 Mb), extensively annotated and amenable to genome editing (3,4). The body is transparent, which enables direct observation of developmental processes and fluorescent reporter constructs (5). Additionally, because *C. elegans* is a facultative outcrosser (males are rare and self-fertile hermaphrodites are the norm), genotypes can be recombined easily using controlled crosses and homogenized by repeated self-crossing. Moreover, strains can be cryopreserved indefinitely (6), which prevents the accumulation of novel mutations and ensures the fidelity of genomes over time. Finally, the other androdioecious *Caenorhabditis* species, *C. briggsae* and *C. tropicalis*, share the advantageous attributes of *C. elegans* and enable comparative studies within the genus.

Comparative studies have informed our understanding of several aspects of *Caenorhabditis* biology, including the evolution of self-fertilization (7,8), the regulation of cell fates involved in vulva development (9–11), the thermotolerance and thermoplasticity of germline development (12), the developmental determinants of sperm size (13), the regulation of dauer formation (14), pheromone signaling and the biosynthesis of pheromones (15), the mutational process (16,17), the abundance and distribution of selfish genetic elements such as transposable elements and toxin-antidote elements (18–21) and the resistance to anthelmintics (22). These investigations have also revealed the changes in overall genome structure, gene structure and protein-coding gene content that are associated with the transition to self-fertilization (23–26), and the strikingly high rates of intrachromosomal rearrangements relative to their outcrossing relatives (27–30).

Despite this rich history of comparative studies, many of the insights from this work are based on just a small number of strains, or even a single strain, within each species. The most common strains used in comparative studies are N2 for *C. elegans*, AF16 for *C. briggsae* and JU1373 for *C. tropicalis*. Although the extensive focus on these particular strains has helped the community standardize experimental procedures and infer biology from the synthesis of independent studies, it also limits our understanding of natural diversity within these species. To help address this limitation, researchers and citizen scientists have isolated many wild *Caenorhabditis* strains from around the globe (31–43). These wild strains represent an enormous reservoir of genetic diversity that can be leveraged to understand the genetic basis of evolutionary processes and trait variation using genome-wide association studies (GWAS). The GWAS approach correlates genotypic variation with phenotypic differences across a set of strains to identify regions of the genome that underlie differences in a trait of interest. In turn, the identified regions, known as quantitative trait loci (QTL), can be dissected to reveal the precise identity of the genetic variants influencing a trait.

In recent years, the genetic and experimental tractability of *C. elegans* has helped it become a leading model for the discovery of genes and variants that underlie quantitative trait variation (44–46). In 2016, we launched the *C. elegans* Natural Diversity Resource (CeNDR) to serve as a public repository for wild *C. elegans* strains, their genome sequences and variant data (47). Since CeNDR was launched, the platform has facilitated approximately 150 studies of natural variation, including the investigation of global population structure (39,41,42), the evolutionary origins of hyper-divergent regions within the genome (41) and the discovery of variants that influence ecologically and biomedically relevant traits using quantitative genetic mappings (35,37,40,48–54). These discoveries highlight the utility of CeNDR as a centralized repository of genetic variation for the *C. elegans* species.

Here, we present the *Caenorhabditis* Natural Diversity Resource (CaeNDR), which enables comparative studies of natural diversity by including 1680 *C. briggsae* and 681 *C. tropicalis* wild strains. The new CaeNDR platform provides three major functions. First, CaeNDR serves as a centralized hub for the collection, maintenance and distribution of strains from the androdioecious *Caenorhabditis* species isolated from nature. Second, CaeNDR leverages recently improved reference genomes for *C. briggsae* and *C. tropicalis* (21,29,55) to provide whole-genome sequence and variant data for strains in the archive. Third, CaeNDR offers web-based tools to help researchers design, perform and analyze GWAS using an augmented version of the NemaScan GWAS pipeline (56). In the following sections, we describe how the major functions of CaeNDR are implemented and explain how the online tools support these functions. The CaeNDR platform can be found at caendr.org.

## Implementation

### A centralized repository for wild strains

CaeNDR provides the *Caenorhabditis* research community with a consolidated collection of wild strains that is continuously updated and rigorously maintained to ensure strain fidelity. We designed the new CaeNDR platform to streamline the submission and acquisition of wild strains. Two options are available for community members that wish to submit strains to CaeNDR. Research groups can contribute strains by completing the Submit a Strain web form or the batch strain submission form, then mailing their strains to CaeNDR. Alternatively, citizen scientists can contribute wild strains by following the collection and processing guidelines at caendr.org/get-involved/citizen-scientists. We aim to preserve the ecological data associated with the wild strains, so we request that contributors include the time, location, photos and descriptions of the landscape and substrate for each strain submission (57). These data, along with attributions for the individuals that sampled and isolated the strains, are added to the CaeNDR database and can be browsed with an interactive map on the website home page. The complete strain dataset is available for download at caendr.org/data/data-release.

Researchers can procure wild strains from CaeNDR individually, or in bulk, using the Request Strains page (caendr.org/request-strains/). Additionally, CaeNDR offers pre-selected sets of strains to facilitate GWAS in each species. These sets include a Divergent Set and several Mapping Sets. The Divergent Set contains 12 strains that are chosen to represent the genetic diversity across a particular species and can be used to assess any heritable trait variation in that subset of strains. If genetic differences among the divergent strains are shown to contribute strongly to trait variation, a GWAS using trait data from mapping sets will likely identify QTL for that trait (46). Each Mapping Set contains 48 strains. Because the statistical power to detect QTL with GWAS increases with the number of strains that are measured, we recommend using at least 96 strains (56).

### A species-wide genomic resource for *C. elegans*, *C. briggsae* and *C. tropicalis*

CaeNDR maintains and regularly updates whole-genome sequence and variant data for strains in the archive. Because selfing *Caenorhabditis* species reproduce predominantly by self-fertilization in the wild, nematodes isolated from the same substrate are often nearly identical, with only a small number of differences attributable to recent, lineage-specific mutations (33,38,39,42,58,59). Therefore, we examine the rates of shared variation among all strains and group strains that are nearly identical into genome-wide haplotypes called isotypes (Supplemental Figure S1). The aligned sequence and variant data for every wild strain that we have sequenced are freely downloadable for each data release. Because strains within isotypes share nearly all variation, their inclusion in a GWAS can lead to false association signals (60). Therefore, the majority of CaeNDR tools, including GWAS, are focused on exploring genetic variation among isotypes. We achieve this focus by choosing a single representative strain for each isotype group, called the isotype reference strain. These strains are selected by choosing the strain from the isotype group with the most sequence coverage. For each species, the single-nucleotide variants (SNVs) and short insertions and deletions (indels) for all isotype reference strains can be downloaded as Variant Call Format (VCF) files (61). CaeNDR also provides additional genomic datasets relevant to the study of natural variation. For example, each of the androdioecious *Caenorhabditis* species carry punctuated regions of extreme sequence divergence that are called hyper-divergent regions (21,41,62). Because these hyper-divergent regions can influence the performance of GWAS and complicate QTL dissection, the physical positions of hyper-divergent regions for all isotype reference strains within *C. elegans* are available for download as Browser Extensible Data (BED) files. In the near future, we will add these datasets for *C. briggsae* and *C. tropicalis*. Importantly, the hyper-divergent regions are identified by extreme sequence divergence relative to the reference genomes for each species, *i.e*. N2 for *C. elegans*, QX1410 for *C. briggsae* and NIC58 for *C. tropicalis*. Therefore, the reference strains will not contain hyper-divergent regions because of reference bias (21,29). The software and methods used to produce all the genomic datasets are available at the Data Release page (caendr.org/data/data-release).

**Figure 1.**
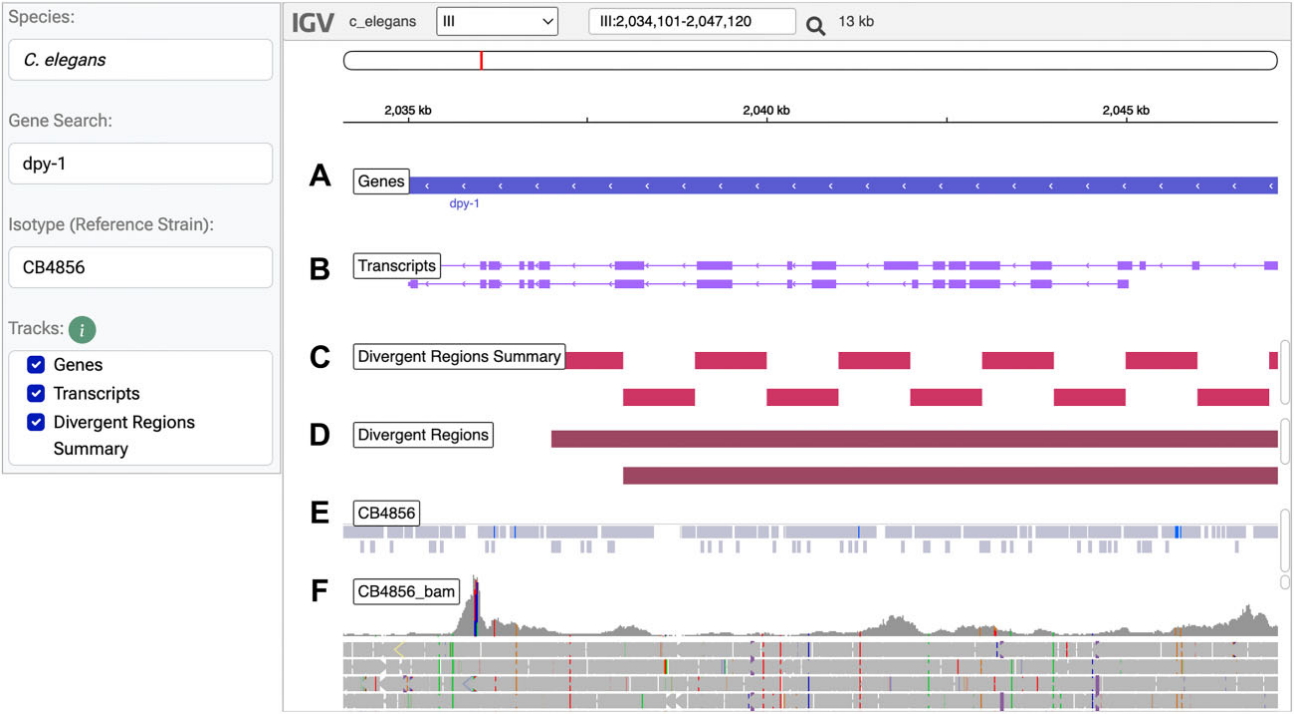
The CaeNDR genome browser. The user specifies the Species in the setup panel to the left. The optional Gene Search field will direct the browser to that gene in the genome (**A**) and display transcripts in the track below (**B**). The Divergent Regions Summary track shows regions of the genome that are classified as hyper-divergent relative to the reference genome in any isotype reference strain within the species (**C**). The Divergent Regions track displays regions that are hyper-divergent relative to the reference genome for individual isotype reference strains, each track feature can be clicked to reveal the isotype reference strain the feature belongs to (**D**). The Isotype field in the setup window on the left is optional and allows users to add VCF and BAM tracks to the browser. The VCF track shows species-wide SNV and short indels in gray, with the variants specific to the isotype reference strain in blue (**E**). The BAM track shows read level data from which the variants were called. The top of the track shows read depth and the bottom shows the paired-end reads (**F**).

The Genome Browser tool (caendr.org/tools/genome-browser/) enables users to visualize the genomic datasets from any of the three species. For example, a user can query a gene or region of interest to visualize genetic variation across all isotype reference strains and inspect the aligned sequencing reads supporting variant calls at that locus. The browser tool uses an embedded JavaScript implementation of the Integrative Genomics Viewer (IGV) (63), which can be configured to show a number of genomic datasets as tracks. By default, the browser displays gene and transcript isoform tracks (Figure 1A, B). Two optional tracks display hyper-divergent regions, either as a species-wide summary or for particular isotype reference strains (Figure 1C, D). The optional VCF track displays high-quality SNVs and indels identified in each isotype reference strain in blue overlaid onto the species-wide variant set in gray (Figure 1E). The optional BAM track shows coverage statistics and paired-end read alignments that give context for how the variants within an isotype reference strain are called (Figure 1F).

To investigate a region further, researchers can use the Variant Annotation tool (caendr.org/tools/variant-annotation), which shows how variants within coding sequences are distributed across the population and supplies predictions for how the variants might impact protein function. The annotation database includes the physical positions of the variants, gene names, names of strains sharing the variants, predicted transcript consequences and protein impacts, among other annotations. Users can filter the database by strain, gene, genetic interval and functional impact class, then browse the filtered database or download it as a Comma-Separated Values (CSV) file for further processing. Importantly, CaeNDR now uses the BCFtools/csq program to predict functional consequences and impacts for variants by considering nearby, *cis-*variants jointly rather than singly when classifying variant consequences (64). To assign variant impacts within transcripts, we group consequences that include changes to amino acids, start and stop codon positions, or splice variants as ‘HIGH’ and all others as ‘LOW’ impact. Beyond this binary impact score, the variant annotation database also includes BLOSUM and Grantham scores, which can help evaluate the potential impact of amino acid substitutions in coding sequences with more nuance (65,66). These scores measure how conserved or radical a particular amino acid substitution is from an evolutionary perspective. The BLOSUM scale ranges from −4 to +11 and the Grantham scale ranges from 5 to 215. Low BLOSUM and high Grantham scores indicate radical amino acid substitutions that are more likely to impact protein function.

### A *Caenorhabditis* GWAS toolbox

CaeNDR hosts several web-based tools to help researchers design, perform and analyze GWAS. A typical GWAS begins with a pilot study to measure a trait of interest across a small number of divergent strains in independent replicates. Researchers then calculate the proportion of trait variance explained by genetic differences among those strains, which is called broad-sense heritability (*H^2^*). In the context of GWAS, the *H^2^* of a trait is useful for determining whether a particular GWAS design will be sufficiently powered to identify QTL. For example, based on simulations in *C. elegans*, 500 strains should be measured in a GWAS to achieve approximately 70% power for detecting a single QTL that underlies a trait with a *H^2^* of 0.2 (56). The Heritability Calculator tool (caendr.org/tools/heritability-calculator) enables users to quickly estimate *H^2^* by submitting data from a pilot experiment as a Tab-Separated Values (TSV) file. The tool reports a *H^2^* estimate along with a plot to help visualize the effect of genetic differences among the strains on the trait. Additionally, the narrow-sense heritability (*h*^2^) is reported, which measures the portion of trait variance explained by additive genetic variance among the strains and is more representative of the heritability that is relevant to GWAS. The heritability report helps users plan the scale of their GWAS and procure the required strains using the Request Strains page. After measuring a trait of interest in a GWAS strain set, mappings can be conducted within CaeNDR’s GWAS mapping portal.

**Figure 2.**
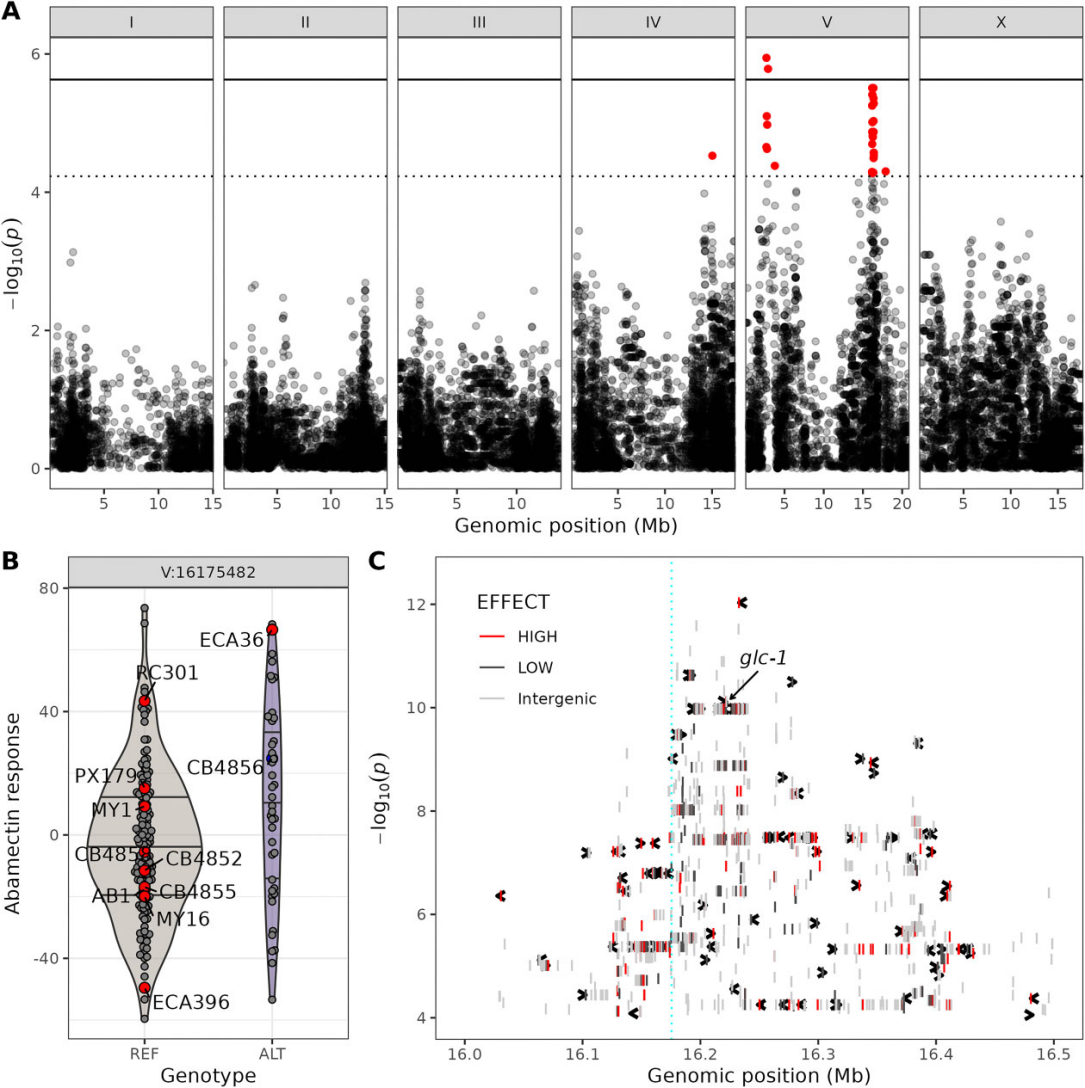
Elements of the abamectin response GWAS mapping report from CaeNDR. (**A**) A Manhattan plot is shown to visualize the significance values for all markers used in the statistical test of association between genotype and phenotype. The y-axis is the negative base 10 log of the *P*-value obtained from the statistical test of association. The x-axis is the genomic position in millions of base pairs and is faceted by chromosome. Markers (SNVs) with a −log_10_*P*-value greater than the Bonferroni-corrected significance threshold (solid line) or the genome-wide eigendecomposition significance threshold (dotted line) are significantly correlated with the phenotype and are colored red. Significant markers denote QTL and indicate that genetic variation linked with the marker could cause differences in the phenotype. (**B**) Violin plots are shown for the most significant marker in the QTL on the right of chromosome V (16.175 Mb). The plot shows the trait values for the strains with the reference (REF) allele compared to the strains with the alternative (ALT) allele for that marker. The abamectin response values shown here are mean centered; the strains with larger values develop better in the presence of abamectin. The horizontal lines indicate the 75th percentile, median and 25th percentile of the data for each genotype. The trait values for some strains are highlighted and labeled because these strains are commonly used to measure dose responses by the community (69–72). A difference in the median trait between the two genotypes is expected for significant markers, and the genotypes of strains at this genomic position can help users plan follow-up experiments to validate the effect of QTL on the trait. (**C**) A fine-mapping plot is shown to visualize the significance of all bi-allelic SNVs in the QTL interval on the right of chromosome V. The x-axis is the physical position in the genome, and the y-axis is the −log_10_*P*-value obtained from a statistical test of association using all SNVs in the interval. The inclusion of all SNVs alters the range of *p*-values relative to GWAS Manhattan plot. We truncated the axes to focus on the center of the QTL. Each SNV is represented by a vertical line and colored by the predicted variant impact on genes: red = HIGH, black = LOW, gray = INTERGENIC. Genes are represented by black arrows showing the direction of the gene and are positioned on the y-axis based on the maximum −log_10_*P*-value of all variants in the gene. The vertical dashed line (cyan) represents the physical position of the most significantly associated SNV identified on the right of chromosome V in the GWAS. The gene *gcl-1* is labeled in the plot because natural variation in this gene was previously shown to confer abamectin resistance (52,68).

The Genetic Mapping tool (caendr.org/tools/genetic-mapping) allows users to perform a GWAS by uploading a TSV file of trait measurements for isotype reference strains. The tool runs a containerized version (hub.docker.com/r/andersenlab/nemascan-nxf) of the NemaScan mapping pipeline (56) to process the trait data and test for correlations between genotype and phenotype at over 50000 marker loci (bi-allelic SNVs) across the *C. elegans*, *C. briggsae* or *C. tropicalis* genomes. The tool produces a detailed mapping report that can be downloaded as an HTML file. The report contains plots and interactive data tables to help users analyze their QTL. To illustrate the utility of the mapping report, we used the Genetic Mapping tool to process data from a previously published GWAS of abamectin responses (67). Abamectin is a widely used agricultural pesticide and the threat of pervasive nematode resistance motivates the search for natural resistance alleles. The Manhattan plot taken from the abamectin response mapping report shows the physical positions of the three QTL identified across the genome (Figure 2A). To visualize the strength of these associations, a phenotype-by-genotype plot for the most significant markers in each QTL is included in the report (Figure 2B). Because long-range linkage disequilibrium (LD) is strong in selfing *Caenorhabditis* species, NemaScan collapses nearby markers with significant correlations into a single QTL (56). Consequently, most QTL identified by NemaScan will contain hundreds of variant sites among the strains in the mapping that could contribute to differences in the trait. Fine mapping tests the association between genotype and phenotype at every SNV in a QTL, not just the marker loci and can help users identify the variants that are most likely to underlie trait differences at a QTL (Figure 2C). In the abamectin example, the gene *gcl-1* is labeled because natural variation in this gene was previously shown to confer abamectin resistance (52,68). Even without this knowledge, the low *P*-value and predicted high effect variant in *glc-1* (arrow, red bar, Figure 2C) would suggest that natural variation in *glc-1* could underlie differences in abamectin responses. Importantly, only two other genes with high-effect variants that have lower *P*-values than *glc-1* were found. This example demonstrates how the list of candidate genes in a region is greatly reduced by cross-referencing the fine-mapping *P*-values and predicted variant impacts provided in the mapping report.

Because many variant sites often exist within QTL and fine mapping sometimes fails to identify obvious candidate genes, each QTL must be validated to test whether specific variants actually cause differences in the trait. Several methods are used to dissect QTL for *Caenorhabditis* (46). A common method used to narrow and validate QTL is the creation and measurement of near-isogenic lines (NILs) (73). A NIL is a strain constructed using genetic crosses that carries a small region from one strain (the donor) introgressed into the genome of another strain (the recurrent parent) (46). Oftentimes, trait differences between the NIL and the recurrent parent are caused by an allelic difference within the introgressed region, validating the effect of the QTL (46). Many quantitative mapping studies in *Caenorhabditis* have successfully leveraged NIL panels to validate and narrow QTL intervals so they contain a small number of potentially causal genes, which were then explicitly tested using CRISPR-Cas9 genome-editing techniques (45). The CaeNDR platform simplifies the construction of NIL panels by providing the Pairwise Indel Finder tool (caendr.org/tools/pairwise-indel-finder) to help users accurately track the introgression of the donor region into the recurrent parent using small indels unique to either genetic background. To use the tool, researchers enter a species, a pair of strains and a specific genomic region. The tool outputs indel variants between 50 and 500 bp that are unique to each strain along with PCR primers that researchers can use to screen progeny for the desired introgression. Additionally, the IGV view supplied by the tool contains a hyper-divergent region track to help avoid indels in hyper-divergent regions because these calls are less reliable and primer searches are more error-prone.

## Applications

### Functional studies of natural variation

Traditional studies of gene function often use loss-of-function or gain-of-function alleles created by mutagenesis to investigate how these changes influence traits (74). However, these mutant alleles might perturb gene function to such an extreme that they prevent researchers from observing traits of interest. To address this challenge, researchers can query genes of interest with the Variant Annotation tool (caendr.org/tools/variant-annotation) to see all of the natural variants, along with any predicted coding-sequence impacts, in a particular genomic region. Because natural variation has vetted these alleles, they are less likely to be highly deleterious and more likely to be ecologically and physiologically relevant. Researchers can then evaluate the influence of these natural variants on traits by introducing them into a genetic background of choice, *e.g*. the *C. elegans* laboratory-adapted strain N2, using outcrossing or genome editing (46).

### Population genomics and adaptive variation

The CaeNDR platform gives researchers the genomic data required to explore how patterns of genetic variation within the three *Caenorhabditis* species have been shaped and maintained by evolutionary processes. For example, an early study of genetic diversity in *C. elegans* identified chromosome-scale selective sweeps that purged genetic diversity in large portions of the global population (32). In agreement with evolutionary theory, the high rate of self-fertilization in *C. elegans* (33,38,58,75) likely exacerbated the effects of selective sweeps (32). However, recent analyses of more geographically representative samples revealed additional structure within the species and relatively high genetic diversity within strains across the Pacific Rim and Hawaiian Islands (39,41,42). Comparatively, *C. briggsae* populations are structured into distinct phylogeographic groups and harbor greater genetic diversity than *C. elegans*; the two primary phylogeographic groups, Temperate and Tropical, are divided along latitudinal lines (62,76–79), which is consistent with differences in thermal tolerance and fitness traits measured in wild strains (12,80). Among the three species, *C. tropicalis* harbors the lowest levels of genetic diversity (81) and appears to be restricted to tropical regions with strong continental population structure and local heterogeneity (21).

These *Caenorhabditis* population genomic studies are useful for several reasons. First, they provide a lens through which researchers can observe how functional variation is shaped and maintained by the effects of mutation, selection, recombination, genetic drift and demography. Beyond contextualizing the adaptive qualities of functional variation, population genomic studies quantify the genetic relatedness among individuals and characterize population structure, which can influence GWAS performance (82). For instance, in *C. elegans*, the optimal GWAS mapping panel is dependent on trade-offs between the amount of natural variation surveyed, the statistical power to detect QTL and the false discovery rate (56). Therefore, as CaeNDR continues to grow, updated population genomic studies will improve the ability to dissect complex traits using quantitative genetics approaches.

### Identifying genotype–phenotype correlations

The primary goal of CaeNDR is to facilitate the identification of genes and genetic variants that drive phenotypic differences in natural populations. CaeNDR’s GWAS mapping tool outputs a detailed report to help users achieve this goal. The report includes Manhattan plots that show the statistical significance of correlations between genetic markers and the variation in the trait of interest across the genome (Figure 2A). Additionally, plots are included to assess the strength of the correlation between genotype and phenotype at the most significant markers in the mapping (Figure 2B). Because most selfing *Caenorhabditis* species have long-range LD, even among chromosomes (62,83,84), the identification of multiple QTL on a single chromosome or even on separate chromosomes could be caused solely by LD. For this reason, the mapping reports show the LD between the most significant markers from each QTL. Another source of spurious associations is population structure among the strains in the mapping population. To help control for false positive associations in the presence of population structure, the NemaScan pipeline uses the resource-efficient linear mixed model approach called fastGWA, which is implemented in the GCTA program (60,85–87). However, strong population structure might still cause spurious associations in GWAS, so we include measures of genomic inflation for users to check for systemic biases in their results. Additionally, users are provided with fine-mapping plots (Figure 2C) and interactive data tables that are useful for narrowing QTL to specific genes and variants that can underlie trait differences. For example, variants that (i) fall within genes, (ii) are strongly associated with trait differences and (iii) are predicted to have high impacts on gene function are good candidates to be tested for their influence on the trait.

## Usage and impact

The CeNDR platform (now CaeNDR) has made a significant scientific impact since its inception seven years ago by supporting nearly 150 publications exploring natural variation. Moreover, the current engagement with CaeNDR is robust, with an average of 490 new visitors per month (average calculated from April 2023 to July 2023) and over 800 registered users in total (July 2023). Registration is free and requires only a valid email address. After registering, users can procure stains, run all tools on the site and easily retrieve data and reports from their personalized results pages.

## Future directions

CaeNDR is updated regularly to incorporate newly isolated wild strains and their genomes. Furthermore, the variant calling pipelines used by CaeNDR will be expanded to incorporate all major classes of structural variants, including deletions, duplications, insertions, inversions and translocations. We also plan to release gene expression data for isotype reference strains, which will enable mediation analyses (88). Additionally, we will continue to release new tools to help mine and explore natural variation across the three species. For example, we are currently developing a phenotype database to store and release trait data that users make public. These data will help the community explore correlations among traits and perform comparative studies. We are also preparing a tool to expedite CRISPR-Cas9 genome editing in wild strains. The tool will output strain specific guide RNA sequences to target edits at user supplied loci, which will simplify the process of testing natural variants for functional effects on *Caenorhabditis* traits.

## Supplementary Material

gkad887_Supplemental_File

## Data Availability

The data underlying this article are available in the Zenodo repository, at https://doi.org/10.5281/zenodo.8393507.
