# Supplementary material for: CaeNDR, the Caenorhabditis Natural Diversity Resource
Source: Nucleic Acids Res. 2023 Oct 19;52(D1):D850–8. doi: 10.1093/nar/gkad887 (PMC10767927; doi:10.1093/nar/gkad887)
Supplement: gkad887_Supplemental_File [file gkad887_supplemental_file.pdf]

Supplemental Materials

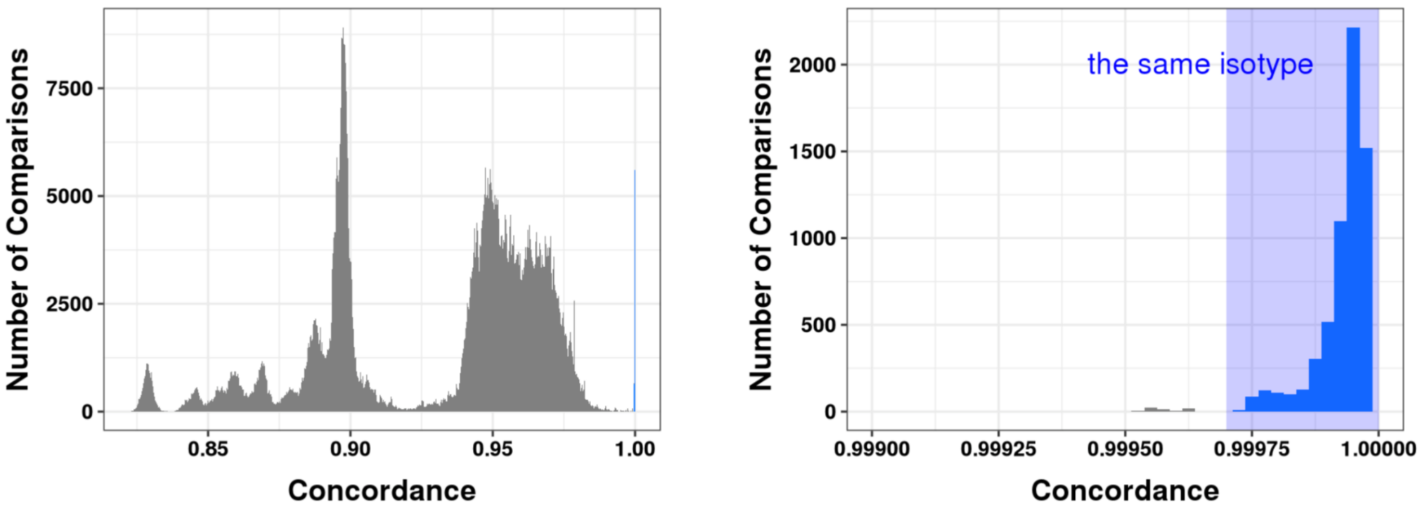

Supplemental Figure 1 - Strain pairwise concordance histogram for *C. elegans*
Concordance analysis is used to group strains that are genetically similar into isotypes. Concordance values for every pair of strains are calculated as the number of shared variant sites divided by the total number of unique variants called within the pair. A strain pair is grouped into the same isotype if the concordance score is greater than 0.9997. The distribution of pairwise concordance values above 0.8 are shown on the left. The plot on the right shows the pairwise concordance values above 0.999. For both histograms the concordance values above 0.9997 are in blue.
